# Effects of acute administration of donepezil or memantine on sleep-deprivation-induced spatial memory deficit in young and aged non-human primate grey mouse lemurs (*Microcebus murinus*)

**DOI:** 10.1371/journal.pone.0184822

**Published:** 2017-09-18

**Authors:** Anisur Rahman, Yves Lamberty, Esther Schenker, Massimo Cella, Solène Languille, Régis Bordet, Jill Richardson, Fabien Pifferi, Fabienne Aujard

**Affiliations:** 1 UMR 7179 Centre National de la Recherche Scientifique, Muséum National d’Histoire Naturelle, Brunoy, France; 2 UCB Pharma s.a., Neuroscience Therapeutic Area, Braine l'Alleud, Belgium; 3 Insitut de Recherches Servier, Croissy-sur-Seine, France; 4 Department of Clinical Pharmacology, Global Clinical Development, Chiesi Farmaceutici, Parma, Italy; 5 Département de Pharmacologie Médicale, Université Lille Nord de France, UDSL, Faculté de Médecine, CHU, Lille, France; 6 Neurosciences Therapeutic Unit, GlaxoSmithKline, Stevenage, Herts., United Kingdom; Nathan S Kline Institute, UNITED STATES

## Abstract

The development of novel therapeutics to prevent cognitive decline of Alzheimer's disease (AD) is facing paramount difficulties since the translational efficacy of rodent models did not resulted in better clinical results. Currently approved treatments, including the acetylcholinesterase inhibitor donepezil (DON) and the N-methyl-D-aspartate antagonist memantine (MEM) provide marginal therapeutic benefits to AD patients. There is an urgent need to develop a predictive animal model that is phylogenetically proximal to humans to achieve better translation. The non-human primate grey mouse lemur (*Microcebus murinus*) is increasingly used in aging research, but there is no published results related to the impact of known pharmacological treatments on age-related cognitive impairment observed in this primate. In the present study we investigated the effects of DON and MEM on sleep-deprivation (SD)—induced memory impairment in young and aged male mouse lemurs. In particular, spatial memory impairment was evaluated using a circular platform task after 8 h of total SD. Acute single doses of DON or MEM (0.1 and 1mg/kg) or vehicle were administered intraperitoneally 3 h before the cognitive task during the SD procedure. Results indicated that both doses of DON were able to prevent the SD-induced deficits in retrieval of spatial memory as compared to vehicle-treated animals, both in young and aged animals Likewise, MEM show a similar profile at 1 mg/kg but not at 0.1mg/kg. Taken together, these results indicate that two widely used drugs for mitigating cognitive deficits in AD were partially effective in sleep deprived mouse lemurs, which further support the translational potential of this animal model. Our findings demonstrate the utility of this primate model for further testing cognitive enhancing drugs in development for AD or other neuropsychiatric conditions.

## Introduction

Alzheimer's disease (AD), the most common form of dementia, is a neurodegenerative disorder clinically characterized by progressive deterioration of cognitive and behavioral function. AD patients exhibit gradual memory and learning impairment, behavioral and personality alterations, and loss of language skills, all of which greatly impairs the individual’s daily functioning and ultimately leading to death [1]. Post-mortem brain sections of AD patients show hallmark histopathological features, including extracellular amyloid-beta (Aß) peptide-containing plaques and intracellular neurofibrillary tangles composed of hyperphosphorylated tau protein [2,3]. While the exact etiology of AD is not yet determined, a cascade of pathophysiological events takes place causing neuronal loss, synaptic dysfunction and neurotransmitter deficiency as the disease progresses. This on-going event impairs crucial memory-related structures, including hippocampus and entorhinal cortex, association cortices and the cerebral default network, causing regional and then diffuse neuronal loss and atrophy [4,5]. This pathological event causes the functional deterioration of neurotransmitter system, leading to a decreased amount of acetylcholine, and activities of choline acetyltransferase (ChAT) and acetylcholinesterase (AChE) in almost the entire neocortex [6]. Impairments in the glutamate neurotransmission system, on the other hand, mediate oxidative stress and excitotoxicity [7,8], resulting in cellular injury and apoptotic cell death.

At present, there are only four FDA-approved, marketed drugs for the symptomatic treatment of AD. Three of these drugs, Donepezil (DON), galantamine and rivastigmine are acetylcholinesterase (AChE) inhibitors and were developed based upon the fact that AD brains show the highest level of cholinergic neuron degeneration in the basal forebrain, resulting in a subsequent reduction in cholinergic transmission to the cerebral cortex [9,10,6]. The fourth drug, memantine (MEM), a low-to-moderate affinity non-competitive antagonist for N-methyl-D-aspartate (NMDA) receptors was developed based upon the observation that soluble Aß oligomers induce memory impairment and synapse loss by NMDA receptor activation [11,12,13]. Despite the evidence for impaired function of other neurotransmitter systems in AD [14,15], findings of reduced cholinergic activity in the basal forebrain-cortical projections in brains of AD patients with cognitive deficits [16] constitute the main rationale of cholinergic replacement therapy as the principal therapeutic approach. The most prescribed drug DON is a highly brain-selective, reversible, competitive AChE inhibitor that has a very prolonged half-life (~70 h) and has been shown to be somewhat effective but quite well tolerated in AD patients [17]. Large-scale clinical studies have reported variable efficacy of DON in mild, moderate and even severe stages of AD based upon cognitive function, daily activities and behavior [18]. In preclinical studies treatment with DON has been shown to improve cognitive performance in several pharmacological models of impaired learning and memory [19,20]. MEM is the only glutamatergic drug approved for the treatment of moderate-to-severe AD patients [21]. There are conflicting results reported regarding the efficacy of MEM treatment. A handful of studies have demonstrated positive results [22,23,24] whereas some other studies reported insignificant or even negative cognitive outcomes [25,26,27]. Despite the reported symptomatic and cognitive benefits of DON and MEM in patients with mild to severe dementia [28,10,29,30], these two drugs neither cure nor prevent progression of the disease and their reported symptomatic benefits could be debated as to whether they reached clinical significance or not [31].

Animal models are essential for investigating the pathophysiological processes underlying AD and the effects of drug therapies. Animal models, especially transgenic AD mouse models provided valuable insight regarding the pathophysiological aspects of the disease but the successful outcome of therapeutic trials based on data generated in these models has so far been lacking [32,33]. For decades researchers had been searching for a valid and more predictive animal model to investigate the disease mechanisms, test treatments and evaluate preventive strategies and cures. Recently, the grey mouse lemur (*Microcebus murinus*), a non-human primate with median survival of 5.7 years for males and a maximum longevity of 12 years, has drawn interest as a potential model for research on ageing (see [34] for extensive review). Age associated functional deficits have been investigated in this species and an age-dependent cerebral atrophy was found to correlate with cognitive impairment. More specifically, the impairment of spatial memory performances was related to the atrophy of the hippocampus and entorhinal cortex in older animals [35]. More recently, it has been demonstrated that about half of the old mouse lemurs displayed a specific deficit in long-term memory retention but not in acquisition in a visual discrimination task [36].

The challenge paradigm, namely sleep-deprivation (SD), used in this study is an established method to induce transient cognitive impairment and has been used in many preclinical studies [37,38,39]. A number of publications reported that this procedure effectively induces temporary cognitive deficits analogous to those shown by patients with AD-like dementia [40]. In a previous study in adult mouse lemurs, we effectively demonstrated the disruptive effects of the SD on spatial memory retrieval, a cognitive function that is affected in AD patients as the disease progresses [41]. SD specifically reduces cortical ACh levels [42], and alters NMDA receptors [43] what may, at least partially, contribute to spatial memory impairment observed after SD. Both observations make this challenge appropriate when targeting an AChE inhibitor such as DON and an NMDA receptors antagonist such as MEM.

We therefore have examined the efficacy of two above-mentioned drugs in the grey mouse lemur, which is phylogenetically proximal to the human species and bears the natural incidence of AD-like pathologies in some aged animals. To our knowledge, there has been no study performed as a back translational experiment combining the effect of age and pharmacotherapy on cognitive function in this novel model. In the current study, we sought to determine the extent to which spatial memory performances would be disrupted by SD challenge in mouse lemurs and also the extent to which an acute pre-treatment of DON or MEM could decrease the negative impact of SD on cognitive processes. Since the disruptive effects of the SD on spatial memory retrieval in our previous study using young animals was effective but not very potent [41], we applied this paradigm in both young and aged animals. Because aged animals have lower performances (more errors) compared to young ones in the spatial memory protocol used in this study [35], we expected a more challenging effect of SD in old animals compared to their young counterparts. Based upon the findings of age-related cognitive research in this primate model, our hypothesis was that DON or MEM would be able to prevent the SD-induced spatial memory deficits.

## Materials and methods

### Ethics statement

All experiments were performed in accordance with the Principles of Laboratory Animal Care (National Institutes of Health publication 86–23, revised 1985) and the European Communities Council Directive (86/609/EEC). The research was conducted under the authorization number 91–305 from the “Direction Départementale de la Protection des Populations” and under the approval of the Cuvier Ethical Committee (Committee number 68 of the "*Comité National de Réflexion Ethique sur l’Expérimentation Animale*") under the authorization number 68–018. In accordance with the recommendations of the Weatherall report, “The use of non-human primates in research”, special attention was paid to the welfare of the animals during this work to minimize nociception [44].

### Animals

Sixty-nine male mouse lemurs were used in these experiments. The experiments were performed in both young (2 to 3 years old) and aged (6 to 7 years old) animals. They were born and raised in the laboratory breeding colony of Brunoy (MNHN, France, license approval N° A91.114.1) from a stock originally derived from the south-western coast of Madagascar 46 years ago. The animals were disease free and the general condition of captivity was maintained under constant temperature of 24–26°C and relative humidity of 55%. Measured food and water were allocated to each animal. The daily food allocation consists of fresh banana, apple and a hand-made mixture of cereals, eggs and milk. Animals were kept in alternating 6-month period of long-days (light:dark 14:10) and short-days (light:dark 10:14). Mouse lemurs were housed in individual cages enriched with tree branches and wooden nest.

### Experimental design

**Fig 1:**
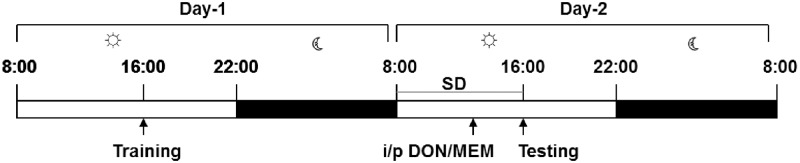
Experimental design including photoperiod. White bar indicates the light-on period and black bar indicate the light-off period. Training started at 16:00 on day 1 and 8h of sleep deprivation (SD) started at 8:00 on day 2, the test was performed immediately after SD on day 2 and DON, MEM or Saline injected 3h before the test.

All the experiments were performed during the long-day photoperiod (lights on at 08:00 and off at 22:00). We induced transient reversible cognitive impairment by 8 h of total SD and spatial memory performance was measured using a circular platform test. In DON experiment 13 young animals (0.1mg/kg, n = 6; 1mg/kg, n = 7) and 13 aged animals (0.1mg/kg, n = 6; 1mg/kg, n = 7) were used. In MEM experiment, 13 young (0.1mg/kg, n = 6; 1mg/kg, n = 7) and 15 aged animals (0.1mg/kg, n = 7; 1mg/kg, n = 8) and in vehicle (Physiological saline) treated experiment 8 young and 6 aged animals were used. All the animals underwent training during day 1 (pre-SD session) and 8h of total SD was performed on day 2 followed by testing immediately after the SD challenge (post-SD session). DON, MEM or saline was injected intraperitoneally 3h before the end of the SD or before the onset of the cognitive function test (Fig 1).

### Circular platform test

Spatial performances were assessed in a circular platform apparatus [35] which is a modified version of Barnes maze especially adapted for mouse lemurs. Briefly, the circular platform is divided into 12 compartments with 12 equally spaced open circular holes (3 cm from perimeter) where a goal box can be affixed for the escape of the animal. The platform is fixed over a spring rotator so it could rotate freely in both directions, to avoid the use of intra-maze cues between successive trials. The whole platform is surrounded by a 15 cm high white wall with a transparent Plexiglas ceiling that allows the mouse lemur to see the extra-maze visual cues. The apparatus is surrounded by a black curtain hung from a square metallic frame, the ceiling of which is a one-way mirror to allow observation for the experimenter. Twelve objects are attached along the inner surface of the frame to serve as visual cues. The starting box is an open-ended dark cylinder positioned in the center of the platform.

In all experiments, training trials (day 1) consisted of 4 trials of maximum 10 min, with an inter-trial interval of 5 min. During the first 2 trials, the animals were habituated in the maze with only one open compartment that contains the goal box and rest of the compartment was closed by thick white paper board. During the third and fourth trial, all the compartments were open and only one compartment gave access to the goal box (the target). Testing consisted of 2 trials of maximum 10 min, in the same condition as the last trials of the training day. Each trial started with the placement of animal in the starting box at the center of the maze. After 60 sec, the box was removed to release the animal. The aim of the tests was to reach the goal box positioned beneath one of the 12 compartments. The position of the target was fixed for each animal throughout the test during day 1 and day 2. When the animal reached the target, the trial was stopped and the animal was allowed to remain in the goal box for 2 min. Performance was assessed by the number of errors (entering the four limbs in an incorrect compartment), the latency (the total time required by the animal to reach the target), the rank of the target zone (two adjacent quadrants surrounding either side of the goal-box containing quadrant; the rank was measured by the number of errors to reach the target zone), and the number of repetition (entry in the same quadrant more than one time) during the testing. Results are expressed by day, each day representing the mean of the two trials of the day.

### SD challenge

Mouse lemurs were subjected to 8h total SD (8:00–16:00) starting at the onset of light period (usual resting phase). The total SD was carried out in the first part of the light period because the sleep is at its maximum during this period [45]. During the whole SD period, mouse lemurs were under constant visual observation in their home cage. The nest and the tree branches were removed from the cage for proper visualization of the animals. SD was achieved by gentle handling, which consists of a standardized procedure of tapping on the cage, moving the index finger in front of the cage and gently shaking the cage if required. Gentle handling was performed if the animal shows signs of sleep such as eye closer, behavioral arrest more than 60 s or huddled body posture. When the above measures were not sufficient to keep the animals awake the front door was opened and closed to stimulate the animals. The electroencephalographic recording confirmed that these interventions keep the animals in a state of wakefulness for 8 h in our previous study [41].

### Drug administration

DON hydrochloride or MEM hydrochloride (generous gift from Dr. Darrel Pemberton, Janssen pharmaceuticals, B-2340, Beerse, Belgium) was dissolved freshly each day in physiological saline to a concentration of 1mg or 10mg/mL (20 μl of tween 80 was added to DON for complete dissolution). DON and MEM (0.1 and 1mg/kg) were injected intraperitoneally (i.p.) to young and aged mouse lemurs 3 h before the end of sleep deprivation and the onset of cognitive function test.

### Rationale for dose choice

DON and MEM doses were chosen to obtain a similar exposure in mouse lemurs as observed at steady state in humans after therapeutic doses. For DON, plasma steady state concentrations of 22.8 and 45.0 ng/mL are reported for 5 and 10 mg/d, respectively [27]. For MEM, plasma steady state concentrations range from 19 to 77 ng/mL after 5 and 20 mg/d, respectively [46,47]. Based on the data from a preliminary pharmacokinetic (PK) study study in grey mouse lemurs, two population PK models were built, one for each drug (not published), using NONMEM 7.2 (Icon Development Solutions, Hanover, Maryland). With these PK models, concentration profiles for different doses of DON and MEM were simulated, eventually leading to the selection of the dose that would satisfy the aforementioned criterion, i.e.0.1 and 1 mg/kg.

### Statistical analysis

For all statistical assessments, data were first assessed for normality using GraphPad Prism software (version 5.01; GraphPad Software Inc. CA, USA). Effect of sessions was evaluated by paired Wilcoxon signed rank test (comparing condition during pre-SD session to condition during post-SD session). To compare the effect of SD between young and aged animals in saline condition, we tested the variation in number of errors between day 1 and day 2 in young *vs* aged animals (number of errors during day 2—number of errors during day 1). Test was performed using a non-parametric Mann-Whitney rank comparison. A p-value <0.05 was considered as significant. All values are given as median and interquartile (IQ: lower quartile–upper quartile) in the text and are represented as box plots in figures.

## Results

### DON effect on SD-induced cognitive deficits in young animals

**Fig 2:**
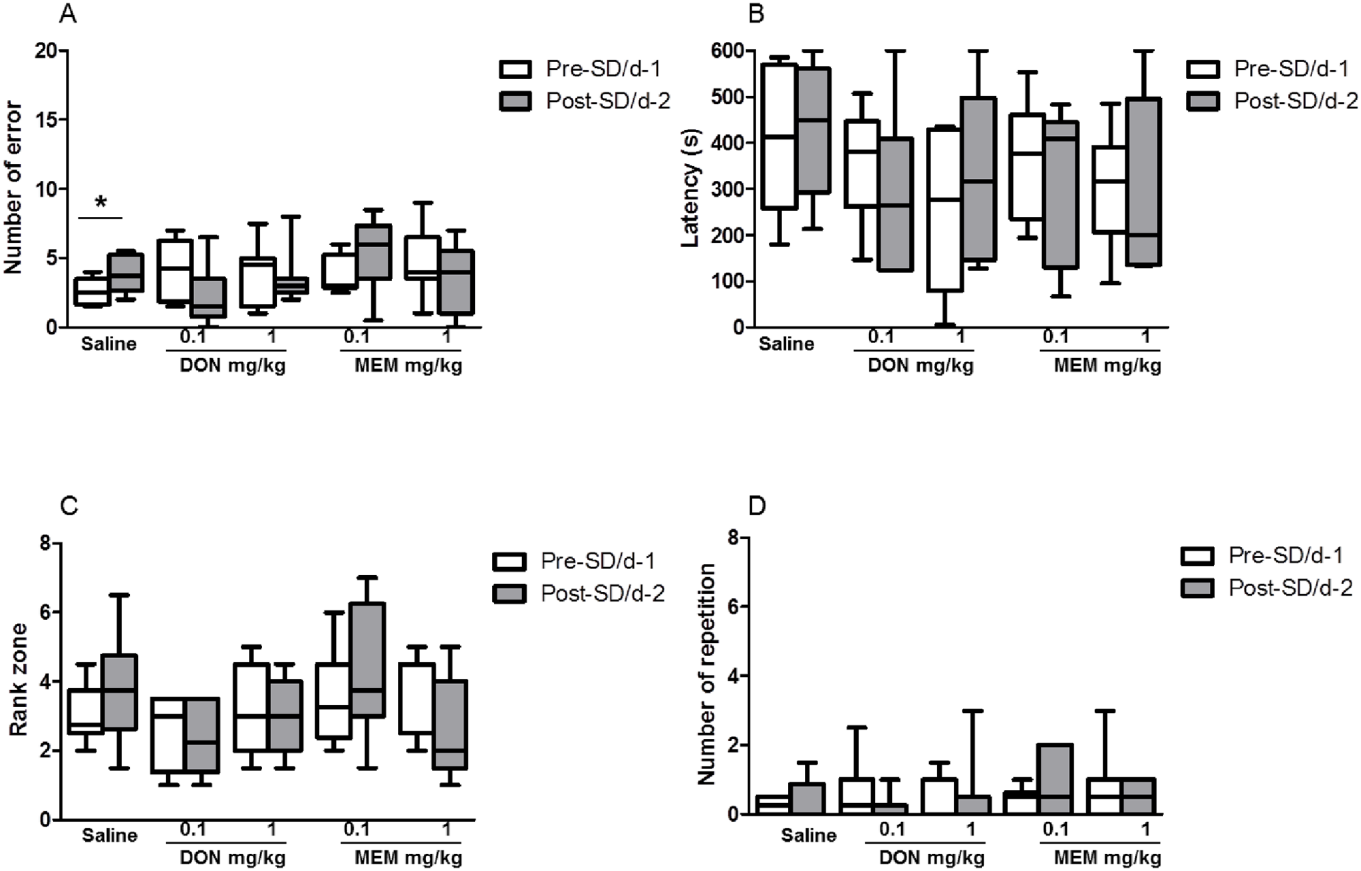
Effects of donepezil (DON) and memantine (MEM) on sleep-deprivation induced spatial memory performances in circular platform test of young grey mouse lemurs showing median. (A) number of errors, (B) latency, (C) rank zone, and (D) number of repetitions. Significant differences for the comparison of day 1 and day 2 (Wilcoxon signed rank test) are indicated as * (p<0.05). Performance was assessed by the number of errors (entering the four limbs in an incorrect compartment), the latency (the total time required by the animal to reach the target), the rank of the target zone (two adjacent quadrants surrounding either side of the goal-box containing quadrant; the rank was measured by the number of errors to reach the target zone), and the number of repetition (entry in the same quadrant more than one time) during the testing.

When the animals under SD were treated with vehicle, they committed significantly more errors during the day 2 trials (testing day) than during the day 1 trials (median: 2.5, IQ: 1.6–3.5 for day 1; median: 3.75, IQ: 2.6–5.3 for day 2, p = 0.021; Fig 2A). This SD-induced increase in the number of errors in the saline group was not observed in the 0.1mg/kg DON injected group (median: 4.25, IQ: 1.88–6.25 for day 1; median 1.5, IQ: 0.75–3.5, p = 0.247) or 1mg/kg DON injected group (median: 4.5, IQ: 1.5–5 for day 1, median: 3, IQ: 2.5–3.5 for day 2, p = 0.734, Fig 2A). Between groups analyses of data for day 1 or day 2 did not show any significant difference. No significant differences were observed for the latency (p = 0.843, Fig 2B), the rank zone (p = 0.232, Fig 2C) and for the number of repetitions (p = 0.824, Fig 2D) between day 1 and day 2 in the saline-treated group. Both doses of DON did not show any significant difference for latency, rank zone or number of errors between day 1 and day 2. Acute injection of DON 0.1 and 1mg/kg prevented the SD-induced retrieval errors.

### MEM effect on SD-induced cognitive deficits in young animals

Fig 2 shows the effects of MEM on spatial memory deficits in young animals. The observed significant increase in the number of errors on day 2 in saline treated animals was prevented by acute treatment with MEM 0.1mg/kg (median: 3, IQ: 2.88–5.25 for day 1; median: 6., IQ: 3.5–7.38 for day 2, p = 0.312; Fig 2A) or 1mg/kg (median: 4, IQ: 3.5–6.5 for day 1; median: 4, IQ: 1–5.5 for day 2, p = 0.552; Fig 2A). Like for the saline treated group, no significant differences were observed in MEM groups for the latency, rank zone or number of repetitions between day 1 and day 2 (Fig 2B–2D). Acute injection of MEM at 0.1 and 1mg/kg prevented the SD-induced retrieval errors.

### DON effect on SD-induced cognitive deficits in aged animals

**Fig 3:**
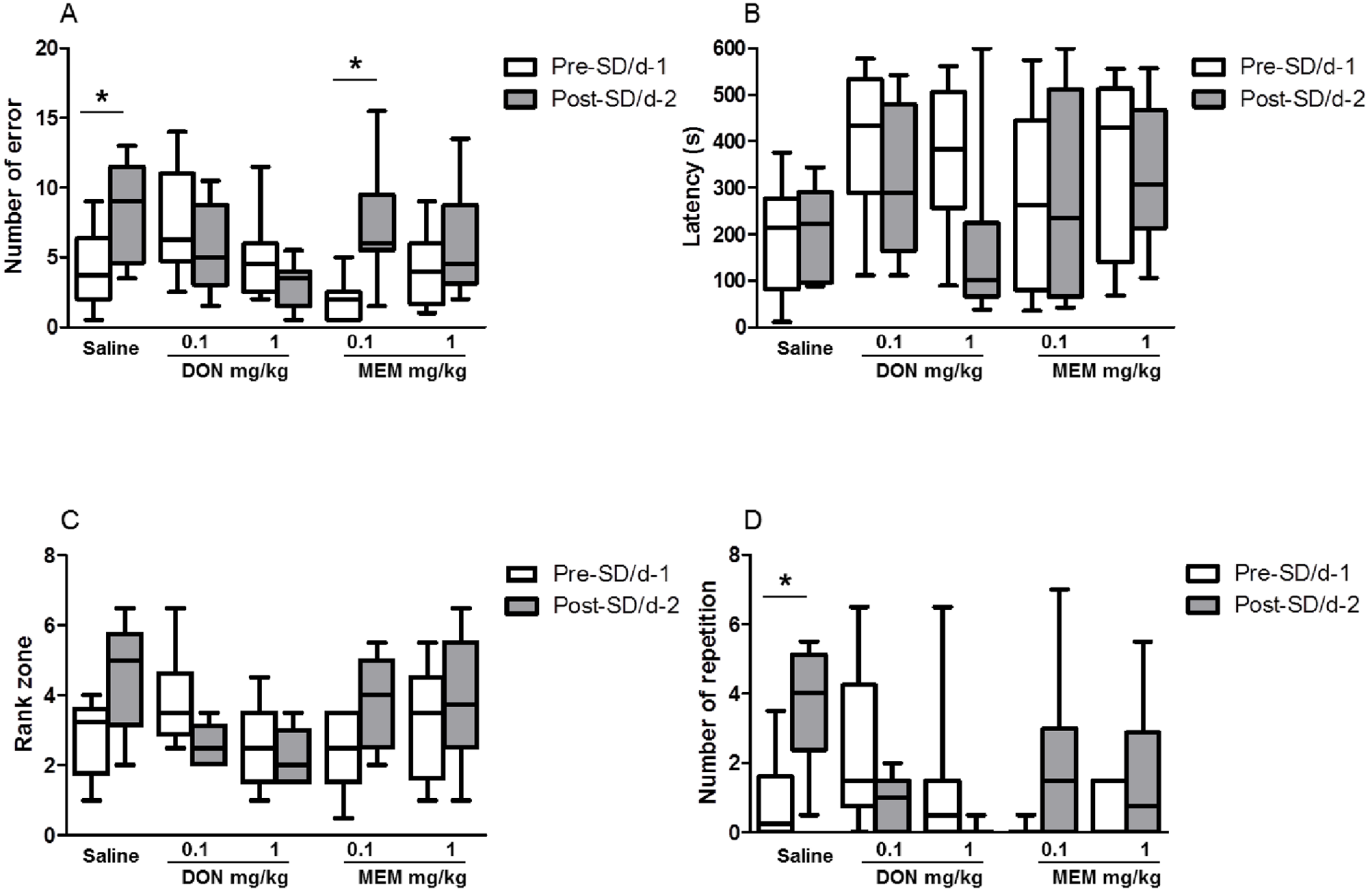
Effects of donepezil (DON) and memantine (MEM) on sleep-deprivation induced spatial memory performances in circular platform test of aged grey mouse lemurs showing median. (A) number of errors, (B) latency, (C) rank zone, and (D) number of repetitions. Significant differences for the comparison of day 1 and day 2 (Wilcoxon signed rank test) are indicated as * (p<0.05). Performance was assessed by the number of errors (entering the four limbs in an incorrect compartment), the latency (the total time required by the animal to reach the target), the rank of the target zone (two adjacent quadrants surrounding either side of the goal-box containing quadrant; the rank was measured by the number of errors to reach the target zone), and the number of repetition (entry in the same quadrant more than one time) during the testing.

As shown in Fig 3A, saline treated-old animals made a significantly higher number of errors during day 2 trials as compared to day 1 trials (median: 3.75, IQ: 2–6.38 for day 1; median: 9, IQ: 4.63–11.50 for day 2, p = 0.035). Analysis of data showed that the number of errors committed during day 2 in the DON groups administered with 0.1mg/kg i (median: 6.25, IQ: 4.75–11 for day 1, median: 5, IQ: 3–8.75 for day 2, p = 0.843) and 1mg/kg i (median: 4.5, IQ: 2.5–6 for day 1, median: 3.5, IQ: 1.5–4 for day 2, p = 0.141) were not significantly different in comparison to day 1. The latency for saline treated- animals and DON treated- animals did not show a significant difference between day 1 and day 2 (saline, p = 0.687; DON 0.1mg/kg, p = 0.437; DON 1mg/kg, p = 0.109; Fig 3B). The number of rank zone in saline-treated animals in day 2 was higher as compared to day 1 and was closed to the chosen level of significance (p = 0.057) whereas the number of rank zone in both doses of DON injected animals was not different between day 1 and day 2 (DON 0.1mg/kg, p = 1.00; DON 1mg/kg, p = 0.141; Fig 3C). The saline-treated old animals showed a significant higher number of repetitions on day 2 as compared to day 1 (p = 0.034). This was not observed in either group of DON injected animals (0.1mg/kg, P = 0.375; or 1mg/kg, p = 0.054; Fig 3D). The acute injection of DON (0.1 or 1mg/kg) was able to prevent SD-induced impairment of memory retrieval in old animals.

### MEM effect on SD-induced cognitive deficits in aged animals

The MEM 0.1mg/kg-injected group also showed a significant higher number of errors at day 2 as compared to day 1 (median: 2, IQ: 0.5–2.5 for day 1; median: 6, IQ: 5.5–9.5 for day 2, p = 0.041). In contrast to vehicle- and MEM 0.1mg/kg- injected groups, the number of errors observed in the MEM 1mg/kg -injected group did not differ between day 1 and day 2 (median: 4, IQ: 1.63–6 for day 1, median: 4.5, IQ: 3.13–8.75 for day 2, p = 0.361). Latency for saline-treated animals and MEM-treated animals did not show any significant difference between day 1 and day 2 (Saline, p = 0.687; MEM 0.1mg/kg, p = 0.932; MEM 1mg/kg, p = 1.00; Fig 3B). For both doses, the number of rank zone of MEM injected animals did not show any significant difference between day 1 and day 2 (MEM 0.1mg/kg, p = 0.149; MEM 1mg/kg, p = 0.611; Fig 3C). Although the number of repetitions at day 2 in MEM 0.1mg/kg injected-animals was not significantly different from day 1, a trend toward a statistically significant effect was noted (p = 0.062, Fig 3D). The number of repetitions in MEM 1mg/kg-injected animals did not differ significantly between day 1 and day 2. The High dose of MEM (1mg/kg) but not the low dose (0.1mg/kg) was noted to prevent the SD-induced impairment of memory retrieval in old animals.

### Comparison of the effects of SD on number of errors between young and aged animals in saline condition

The variation in number of errors between day 1 and day 2 is significantly increased in aged animals median: 7.0, IQ: 2.5–7.5) compared to young (median: 1.0, IQ: 0.5–1.75) (p = 0.0011).

## Discussion & conclusion

In a previous study we demonstrated that 8h of SD by gentle handling was a valid paradigm to induce a transient impairment of spatial memory performances in young mouse lemurs [41]. The present study confirms our former findings and demonstrates that SD-induced spatial memory impairment can be reversed in both young and aged animals by an acute administration of DON and MEM.

The current treatments of AD are designed either to augment the cholinergic function by inhibiting the enzyme AChE or to prevent the excitatory effect of NMDA receptors. A number of preclinical studies have reported cognition-enhancing effects of DON [48,49,50,51] following chronic administration in mouse models of AD. Few preclinical studies have tested the effects of acute administration of DON or MEM on memory performances [52,53]. Our present study is one of the first to test the acute effect of these clinically used drugs in a model which is phylogenitically proximal to human and an important natural model relevant for aging or neurodegenerative diseases.

We believe that our challenge paradigm of SD by gentle handling is a valid protocol for producing a transient reversible cognitive impairment without causing much stress. SD procedures in rodents are normally performed either by moving treadmills or rotating wheels or "disk-over-water" method in which the method itself incurs a significant amount of stress in addition to SD challenge. Moreover, we have successfully established this challenge paradigm to induce a spatial memory impairment in a previous study in grey mouse lemurs [41] and electroencephalographic recordings have shown that this intervention effectively maintains the animals in a state of wakefulness for several hours both in mouse lemurs [41] and rats [54], without substantial changes in serum cortisol level in mice [55]. Together, our data support the utility of SD as a suitable paradigm for the induction of a cognitive impairment not only in young animals but also in aged animals.

In the present study, DON was shown to be effective in young and aged animals; at both low (0.1mg/kg) and high (1mg/kg) doses, DON was able to prevent the significant increase of SD-induced spatial memory retrieval errors as compared to vehicle treated animals (Figs 2A and 3A). Our finding is consistent with the finding of [56] indicating that DON at doses of 0.5 and 1 mg/kg was effective in reversing scopolamine-induced spatial memory performances in rats submitted to a water maze task. By and large, DON has shown to reverse scopolamine-induced learning deficits in reference and working memory tests in rodents [57,20,58]. Interestingly, a study performed in transgenic AD mice revealed that sub-chronic administration of DON for 2 weeks concomitantly improved the cognitive deficits along with the dose-dependent decrease of brain soluble and insoluble Aβ40 and 42 [59]. Consistent with preclinical studies, DON has been shown to counteract the negative impact of SD on cognitive function in a group of healthy individuals whose cognitive performance was greatly impaired by SD [60,61]. Previous studies using cholinomimetics and AChE inhibitors have reported that these drugs were able to improve memory function at low doses but higher doses were shown to impair memory function, thereby resulting in an inverted U-shaped dose-response curve [62,63]. Despite the fact that we only used two doses, in the present study, we did not find this bell-shaped curve since both the low and high doses were able to reduce the SD-induced memory impairment in young and aged animals.

The excitatory amino acid, glutamate, acts through NMDA receptors and these receptors play an important role in calcium homeostasis, synaptic plasticity, and learning and memory [64]. Dysregulation of NMDA receptors have been found in AD, and it has been reported that NMDA receptor-expressing neurons are more vulnerable to AD-related insults [65,66]. A number of studies have examined the effect of MEM on cognitive functions following different protocols in different animal models [48,49,67] and most of them reported positive effects of MEM on cognitive function. In the present study, acute treatment with MEM at 0.1 and 1mg/kg improved the memory retrieval performances in young animals that were perturbed by a SD challenge. However, in aged animals, MEM failed to improve the spatial memory performances induced by SD challenge at 0.1 mg/kg. Only the dose of 1 mg/kg successfully reversed the SD-induced memory retrieval impairment. This suggests that the SD paradigm is more challenging for aged animals compared to young ones. It is worth mentioning here that low doses of MEM have been found to impair the retrieval memory after 24 h of learning in adult rat [26]. Interestingly, [50] showed that acute treatment with MEM improved working and spatial memory dysfunction in transgenic AD mice, which is in line with our findings. Also consistent with our results, a recent study by [68] investigated the effect of MEM on SD-induced cognitive impairment in *Octodon degus*, a rodent model that exhibits a natural occurrence of some AD-related neuropathologies. Their findings indicated that MEM was able to prevent reference and working memory impairment caused by SD in both young and aged animals. Several clinical studies in healthy volunteers have examined the effect of MEM after a single dose on mood, attention, immediate or delayed verbal memory and visuospatial memory and reported either weak positive effect or a negative impact depending on the tasks used [69,70,71]. In contrast, some clinical studies in patients with moderate to severe AD demonstrate that MEM was able to slow cognitive decline [72,73].

From a pharmacokinetic perspective, two possible limitations to the interpretation of the results are worth mentioning. The first is that the PK modelson which the doses were chosen were built from PK data obtained in young adults: it is possible that age may affect the pharmacokinetics of the drugs. Hence, the same dose could in theory give different concentration levels in young and aged mouse lemurs (and therefore could explain, at least in part, the lack of efficacy in spatial memory performance shown by MEM at the lower dose). The second limitation is the lack of information available regarding the distribution properties of DON and MEM in mouse lemurs, and especially the drug penetration into the brain. Doses for both compounds were chosen to reflect plasma steady state concentrations observed in humans, but even when plasma concentrations match perfectly across species, it is not possible to infer the amount of drug actually reaching AChE enzymes and NMDA receptors in the brain. With regard to these limitations, further and specific studies are required. It is worth mentioning here that we avoided sampling blood during the sleep deprivation procedure to avoid unnecessary stress to the mouse lemurs that could hamper the cognitive performance of the animals.

To obtain the optimum translational efficacy, a novel therapeutic agent should be tested in an animal model that has a satisfactory level of construct, face and predictive validity. Clinical efficacy and drug toxicity translated from preclinical rodent models to humans has not always resulted in a reliable degree of predictability with regard to clinical outcome. If transgenic mouse models of AD have been providing invaluable information regarding molecular and pathophysiological aspects of the disease, their translational efficacy has been to date disappointing. Independent of the problem of underpowered studies with a too small number of animals that may partly explain the lack of translatability noted when one use transgenic mice relevant for AD, an ideal preclinical approach should select the most relevant animal model that can provide a reflective behavioural task measuring higher-level cognitive functions in order to achieve the highest translational efficacy. Considering the complexity of the human brain, and the high genetic homology between non-human primates and human species, the non-human primate mouse lemurs may serve as a potential model for evaluating cognition-enhancing therapeutic agents. In terms of construct validity, some aged mouse lemurs show most of the AD-related neuropathological hallmarks as demonstrated by pathological studies on brain section [74]. In our previous study we demonstrated that spatial memory performances could be impaired transiently in young animals by SD [41], which was more challenging in aged animals as suggested by the present study. Indeed, SD impacts more aged animals as revealed by their higher increase in number of errors between day 1 and day 2 compared to young. Moreover, [35] and [75], using magnetic resonance imaging and behavioural studies, recently demonstrated that both executive function and spatial memory decline with age in this primate. This provides partial face validity of this animal model. The interest of using non-human primates to obtain the optimum translational efficacy is supported by similar studies in other non-human primates. In the study by [76], testing the effects of memantine and galantamine on cognitive performances in aged rhesus macaques, the authors report mild beneficial effects on some aspects of cognitive performance in aged animals. This observation is in accordance with the present results and in agreement with the human observations with these drugs, but in contrast to the more positive effects reported in the rodent literature. These data suggest that the nonhuman primate might have more predictive validity for drug development in this area than comparable rodent assays. This higher predictive validity can be explained by the closer phylogenic proximity of primates, but also maybe by the higher inter-individual variability that characterizes primates and may help mimic better human variability.

In conclusion, the present study, to our knowledge, is the first in which acute administration of t two approved drugs for treating cognitive function in AD patients has been shown to improve spatial memory impairment produced by a sleep deprivation procedure in the non-human primate grey mouse lemur. Although the SD challenge could not induce a learning-as opposed to retrieval- deficit in spatial memory [41], the symptomatic benefit observed after a single administration of DON or MEM in the present study give more confidence in the predictive validity of this model. Further studies are required to achieve a higher level of validation by including the testing of other cognitive domains and the use of different challenges to induce transient cognitive impairment and understand the molecular and cellular mechanisms subserving these alterations.

## Supporting information

S1 Table Supplementary table 1. Raw data of young and aged animals before (day 1, D1) and after (day 2, D2) 8h sleep deprivation, with saline or two doses (0.1mg/kg [0.1] or 1 mg/kg [1]) of donepezil (DPZ) or memantine (MEM).
(DOCX)

## References

[1] Selkoe D.J. Alzheimer’s disease: genes, proteins and therapy. Physiol. Rev. 2001; 81:741–766. 11274343 10.1152/physrev.2001.81.2.741

[2] Hardy J.A., Higgins G.A. Alzheimer’s disease: the amyloid cascade hypothesis. Science. 1992; 256: 184–185. 1566067 10.1126/science.1566067

[3] Blennow K., De Leon M.J., Zetterberg H. Alzheimers disease. Lancet. 2006; 368: 387–403. doi: 10.1016/S0140-6736(06)69113-7 16876668 10.1016/S0140-6736(06)69113-7

[4] Selkoe D., Schenk D.. Alzheimer's disease: molecular understanding predictsamyloid-based therapeutics. Annu. Rev. Pharmacol. Toxicol. 2003; 43: 545–584. doi: 10.1146/annurev.pharmtox.43.100901.140248 12415125 10.1146/annurev.pharmtox.43.100901.140248

[5] Greicius M.D., Srivastava G., Reiss A. L., Menon V. Default-mode network activity distinguishes Alzheimer's disease from healthy aging: evidence from functional MRI. Proc. Natl. Acad. Sci. U. S. A. 2004; 101(13): 4637–4642. doi: 10.1073/pnas.0308627101 15070770 10.1073/pnas.0308627101 PMC384799

[6] Coyle J.T., Price D.L., DeLong M.R. Alzheimer’s disease: a disorder of cortical cholinergic innervation. Science. 1983; 219: 1184–1190. 6338589 10.1126/science.6338589

[7] Hynd M.R., Scott H. L., Dodd P. R. Glutamate-mediated excitotoxicity and neurodegeneration in Alzheimer's disease. Neurochem. Int. 2004; 45(5): 583–595. doi: 10.1016/j.neuint.2004.03.007 15234100 10.1016/j.neuint.2004.03.007

[8] Shah R. S., Lee H. G., Xiongwei Z., Perry G., Smith M. A., Castellani R. J. Current approaches in the treatment of Alzheimer's disease. Biomed. Pharmacother. 2008; 62(4): 199–207. doi: 10.1016/j.biopha.2008.02.005 18407457 10.1016/j.biopha.2008.02.005

[9] Davies P., Maloney A.J. Selective loss of central cholinergic neurons in Alzheimer’s disease. Lancet 1976; 2(8000): 1403. 10.1016/s0140-6736(76)91936-x 63862

[10] Lanctot K.L., Herrmann N., Yau K.K., Khan L.R., Liu B.A., LouLou M.M., Einarson T.R. Efiicacy and safety of cholinesterase inhibitors in Alzheimer’s disease: a meta-analysis. C.M.A.J. 2003; 169(6): 557–564. PMC191283 12975222

[11] Shankar G.M., Bloodgood D.L., Townsend M., Walsh D.M., Selkoe D.J., Sabatini B.L. Natural oligomers of the Alzheimer amyloid-beta induce reversible synapse loss by modulating an NMDA-type glutamate receptor-dependant signalling pathway. J. Neurosci. 2007; 27: 2866–2875. doi: 10.1523/JNEUROSCI.4970-06.2007 17360908 10.1523/JNEUROSCI.4970-06.2007 PMC6672572

[12] Lacor P.N., Buniel M.C., Furlow P.W., Clemente A.S., Velasco P.T., Wood M., Viola K.L., Klein W.L. A beta-oligomer induced abberation in synapse composition, shape, and density provide a molecular basis for loss of connectivity in Alzheimer’s disease. J. Neurosci. 2007; 27: 796–807. doi: 10.1523/JNEUROSCI.3501-06.2007 17251419 10.1523/JNEUROSCI.3501-06.2007 PMC6672917

[13] Lesne S., Koh M.T., Kotilinek L., Kayed R., Kayed R., Glabe C.G., Yang A., Gallagher M., Ashe K.H. A specific amyloid-beta protein assembly in the brain impairs memory. Nature. 2006; 440: 352–357. doi: 10.1038/nature04533 16541076 10.1038/nature04533

[14] Davies P., Wolozin B.L. Recent advances in the neurochemistry of Alzheimer’s disease. J. Clin. Psychiatry 1987; 48: 23–30. 2883177

[15] Strong R. Neurochemical changes in the aging human brain: implications for behavioural impairment and neurodegenerative disease. Geriatrics. 1998; 53: S9–12. 9745628

[16] Auld D. S., Kornecook T. J., Bastianetto S., Quirion R. Alzheimer's disease and the basal forebrain cholinergic system: relations to beta-amyloid peptides, cognition, and treatment strategies. Prog. Neurobiol. 2002; 68(3): 209–245. 12450488 10.1016/s0301-0082(02)00079-5

[17] Prvulovic D., Schneider B. Pharmacokinetic and pharmacodynamic evaluation of donepezil for the treatment of Alzheimer’s disease. Expert Opin. Drug Metab. Toxicol. 2014; 10:1039–1050. doi: 10.1517/17425255.2014.915028 24785550 10.1517/17425255.2014.915028

[18] Birks J., Harvey R.J. Donepezil for dementia due to Alzheimer’s disease. Cochrane Database Syst. Rev. 2006; CD001190 doi: 10.1002/14651858.CD001190.pub2 16437430 10.1002/14651858.CD001190.pub2

[19] Li Q., Chen M., Liu H., Yang L., Yang G. Expression of APP, BACE1, AChE and ChAT in an AD model in rats and the effect of donepezil hydrochloride treatment. Mol. Med. Rep. 2012; 6(6):1450–1454. doi: 10.3892/mmr.2012.1102 23023803 10.3892/mmr.2012.1102

[20] Ogura H., Kosasa T., Kuriya Y., Yamanishi Y. Donepezil, a centrally acting acetyl ChE inhibitor, alleviates learning deficits in hypocholinergic models in rats. Methods Find. Exp. Clin. Pharmacol. 2000; 22: 89–95. 10849891 10.1358/mf.2000.22.2.796070

[21] Kurz A., Grimmer T. Efficacy of memantine hydrochloride once-daily in Alzheimer’s disease. Expert Opin. Pharmacother. 2014; 15(13): 1955–1960. doi: 10.1517/14656566.2014.945907 25085661 10.1517/14656566.2014.945907

[22] McShane R., Areosa Sastre A., Minakaran N. Memantine for dementia. Cochrane Database Syst. Rev.2006; CD003154 doi: 10.1002/14651858.CD003154.pub5 16625572 10.1002/14651858.CD003154.pub5

[23] Bakchine S., Loft H. Memantine treatment in patients with mild to moderate Alzheimer’s disease: results of a randomised, double-blind, placebo-controlled 6 month study. J. Alzheimers Dis. 2007; 11:471–479. 17656827 10.3233/jad-2007-11409

[24] Minkeviciene R., Banerjee P., Tanila H. Memantine improves spatial learning in a transgenic mouse model of Alzheimer’s disease. J. Pharmacol. Exp. Ther. 2004; 311: 677–682. doi: 10.1124/jpet.104.071027 15192085 10.1124/jpet.104.071027

[25] Vercelletto M., Boutoleau-Bretonnière C., Volteau C., Puel M., Auriacombe S., Sarazin M., Michel B.F., Couratier P., Thomas-Antérion C., Verpillat P., Gabelle A., Golfier V., Cerato E., Lacomblez L; French research network on Frontotemporal dementia. Memantine in behavioural variant fronto temporal dementia. J. Alzheimers Dis. 2011; 23(4): 749–759. doi: 10.3233/JAD-2010-101632 21157021 10.3233/JAD-2010-101632

[26] Creeley C., Wozniak D.F., Labruyere J., Taylor G.T., Olney J.W. Low doses of memantine disrupt memory in adult rats. J. Neurosci. 2006; 26: 3923–3932. doi: 10.1523/JNEUROSCI.4883-05.2006 16611808 10.1523/JNEUROSCI.4883-05.2006 PMC6673894

[27] Repantis D., Laisney O., Heuser I. Acetylcholinesterase inhibitors and memantine for neuroenhancement in healthy individuals: A systemic review. Pharmacol. Res. 2010; 61: 473–481. doi: 10.1016/j.phrs.2010.02.009 20193764 10.1016/j.phrs.2010.02.009

[28] Geldmacher D.S. Long-term cholinesterase inhibitor therapy for Alzheimer’s disease: practical considerations for the primary care physician. Prim. Care Companion J. Clin. Psychiatry. 2003; 5(6): 251–259. 15213795 10.4088/pcc.v05n0602 PMC419395

[29] Burns A., Rossor M., Hecker J., Gauthier S., Petit H., Moller H.J., Rogers S.L., Friedhoff L.T. The effects of donepezil in Alzheimer’s disease results from a multinational trial. Dement. Geriatr. Cogn. Disord. 1999; 10(3): 237–244. doi: 17126 10325453 10.1159/000017126

[30] Gauthier S., Loft H., Cummings J. Improvement in behavioural symptoms in patients with moderate to severe Alzheimer’s disease by memantine: a pooled data analysis. Int. J. Geriatr. Psychiatry. 2008; 23(5): 537–545. doi: 10.1002/gps.1949 18058838 10.1002/gps.1949

[31] Raina P., Santaguida P., Ismaila A., Patterson C., Cowan D., Levine M., Booker L., Oremus M. Effectiveness of cholinesterase inhibitors and mematine for treating dementia: evidence review for a clinical practice guideline. Ann. Intern. Med. 2008; 148(5): 379–397. 18316756 10.7326/0003-4819-148-5-200803040-00009

[32] Windisch M. We can treat Alzheimer’s disease successfully in mice but not in men: failure in translation? A perspective. Neurodegener. Dis. 2014; 13(2–3): 147–150. doi: 10.1159/000357568 24401335 10.1159/000357568

[33] Laurijssens B., Aujard F., Rahman A. Animal model of Alzheimer’s disease and drug development. Drug Discov. Today Technol. 2013; 10(3): e319–27 doi: 10.1016/j.ddtec.2012.04.001 24050129 10.1016/j.ddtec.2012.04.001

[34] Languille S., Blanc S., Blin O., Canale C.I., Dal-Pan A., Devau G., Dhenain M., Dorieux O., Epelbaum J., Gomez D., Hardy I., Henry P.Y., Irving E.A., Marchal J., Mestre-Francés N., Perret M., Picq J.L., Pifferi F., Rahman A., Schenker E., Terrien J., Théry M., Verdier J.M., Aujard F. The grey mouse lemur: a non-human primate model for ageing studies. Ageing Res. Rev. 2012; 11(1): 150–162. doi: 10.1016/j.arr.2011.07.001 21802530 10.1016/j.arr.2011.07.001

[35] Picq J.L., Aujard F., Volk A., Dhenain M. Age-related cerebral atrophy in non-human primates predicts cognitive impairments. Neurobiol. Aging. 2012; 33(6): 1096–1109. doi: 10.1016/j.neurobiolaging.2010.09.009 20970891 10.1016/j.neurobiolaging.2010.09.009 PMC3381737

[36] Picq JL., Villain N., Gary C., Pifferi F., Dhenain M. Jumping stand apparatus reveals rapidly specific age-related cognitive impairments in mouse lemur primates. PLoS One. 2015; 10(12): e0146238 doi: 10.1371/journal.pone.0146238 26716699 10.1371/journal.pone.0146238 PMC4696676

[37] Graves L.A., Heller E.A., Pack A.I., Abel T. Sleep deprivation selectively impairs memory consolidation for contextual fear conditioning. Learn. Mem. 2003; 10: 168–176. doi: 10.1101/lm.48803 12773581 10.1101/lm.48803 PMC202307

[38] Kim E.Y., Mahmoud G.S., Grover L.M. REM sleep deprivation inhibits LTP in vivo in area CA1 of rat hippocampus. Neurosci. Lett. 2005; 388: 163–167. doi: 10.1016/j.neulet.2005.06.057 16039776 10.1016/j.neulet.2005.06.057

[39] Colavito V., Fabene P.F., Grassi-Zucconi G., Pifferi F., Lamberty Y., Bentivoglio M., Bertini G. Experimental sleep deprivation as a tool to test memory deficits in rodents. Front. Syst. Neurosci. 2013; doi: 10.3389/fnsys.2013.00106 24379759 10.3389/fnsys.2013.00106 PMC3861693

[40] Alzoubi K.H., Khabour O.F., Rashid B.A., Damaj I.M., Salah H.A. The neuroprotective effect of vitamin E on chronic sleep deprivation-induced memory impairment: the role of oxidative stress. Behav. Brain Res. 2012; 226(1): 205–210. doi: 10.1016/j.bbr.2011.09.017 21944940 10.1016/j.bbr.2011.09.017

[41] Rahman A., Languille S., Lamberty Y., Babiloni C., Perret M., Bordet R., Blin O., Jacob T., Auffret A., Schenker E., Richardson J., Pifferi F., Aujard F. Sleep deprivation impairs spatial retrieval but not spatial learning in the non-human primate grey mouse lemur. PLoS One 2013; doi: 10.1371/journal.pone.0064493 23717620 10.1371/journal.pone.0064493 PMC3661499

[42] Boonstra T.W., Stins J.F., Daffertshofer A., and Beek P.J. Effects of sleep deprivation on neural functioning: an integrative review. Cell Mol Life Sci. 2007; 64(7–8): 934–946. doi: 10.1007/s00018-007-6457-8 17347797 10.1007/s00018-007-6457-8 PMC2778638

[43] Xie M., Yan J., He C., Yang L., Tan G., Li C., Hu Z., Wang J. Short-term sleep deprivation impairs spatial working memory and modulates expression levels of ionotropic glutamate receptor subunits in hippocampus. Behav Brain Res. 2015; 286: 64–70. doi: 10.1016/j.bbr.2015.02.040 25732956 10.1016/j.bbr.2015.02.040

[44] Weatherall, FRS. D. The use of non-human primate in research, The Weatherall report. 2006.

[45] Pifferi F., Rahman A., Languille S., Auffret A., Babiloni C., Blin O., Lamberty Y., Richardson J.C., Aujard F. Effects of dietary resveratrol on the sleep-wake cycle in the non-human primate grey mouse lemur (*Microcebus murinus*). Chronobiol. Int. 2012; 29(3): 261–270. doi: 10.3109/07420528.2011.654019 22390239 10.3109/07420528.2011.654019

[46] Gomolin I.H., Smith C., Jeitner T.M. 2010 Once-daily memantine: pharmacokinetic and clinical considerations. J. Am. Geriatr. Soc. 58(9), 1812–1813. doi: 10.1111/j.1532-5415.2010.03048.x 20863351 10.1111/j.1532-5415.2010.03048.x

[47] Periclou A., Ventura D., Rao N., Abramowitz W. Pharmacokinetic study of memantine in healthy and renally impaired subjects. Clin. Pharmacol. Ther. 2006; 79(1): 134–143. doi: 10.1016/j.clpt.2005.10.005 16413248 10.1016/j.clpt.2005.10.005

[48] Umukoro S., Adewole F.A., Eduviere A.T., Aderibigbe A.O., Onwuchekwa C. Free radical scavenging effect of donepezil as the possible contribution to its memory enhancing activity in mice. Drug Res. (Stuttg). 2014; 64(5): 236–239. 24203083 10.1055/s-0033-1357126

[49] Xia Z., Zhang R., Wu P., Xia Z., Hu Y. Memory defect induced by ß-amyloid plus glutamate receptor agonist is alleviated by catalpol and donepezil through different mechanisms. Brain Res. 2012; 1441: 27–37. doi: 10.1016/j.brainres.2012.01.008 22305339 10.1016/j.brainres.2012.01.008

[50] Martinez-Coria H., Green K.N., Billings L.M., Kitazawa M., Albrecht M., Rammes G., Parsons C.G., Gupta S., Banerjee P., LaFerla F.M. Memantine improves cognition and reduces Alzheimer’s-like neuropathology in transgenic mice. Am. J. Pathol. 2010; 176: 870–880. doi: 10.2353/ajpath.2010.090452 20042680 10.2353/ajpath.2010.090452 PMC2808092

[51] Scholtzova H., Wadghiri Y.Z., Douadi M., Sigurdsson E.M., Li Y.S., Quartermain D., Banerjee P., Wisniewski T. Memantine leads to behavioral improvement and amyloid reduction in Alzheimer’s-disease-model transgenic mice shown as by micromagnetic resonance imaging. J. Neurosci. Res. 2008: 224; 57–60. 10.1002/jnr.21713 PMC2723808 18615702

[52] Nagakura A., Shitaka Y., Yarimizu J., Matsuoka N. Characterization of cognitive deficits in a transgenic mouse model of Alzheimer’s disease and effects of donepezil and memantine. Eur. J. Pharmacol. 2013; 703(1–3): 53–61. doi: 10.1016/j.ejphar.2012.12.023 23276665 10.1016/j.ejphar.2012.12.023

[53] Ihalainen J., Sarajarvi T., Rasmusson D., Kemppainen S., Keski-Rahkonen P., Lehtonen M., Banerjee P.K., Semba K., Tanila H. 2011. Effects of memantine and donepezil on cortical and hippocampal acetylcholine levels and object recognition memory in rats. Neuropharmacology. 2011; 61(5–6): 891–899. doi: 10.1016/j.neuropharm.2011.06.008 21704049 10.1016/j.neuropharm.2011.06.008

[54] Tobler I., Borbély A.A. The effect of 3-h and 6-h sleep deprivation on sleep and EEG spectra of the rat. Behav. Brain Res. 1990; 36(1–2): 73–78. 2302323 10.1016/0166-4328(90)90161-7

[55] Hagewoud R., Havekes R., Tiba P.A., Novati A., Hogenelst K., Weinreder P., Van der Zee E.A., Meerlo P. Coping with sleep deprivation: shifts in regional brain activity and learning strategy. Sleep. 2010; 33(11): 1465–1473. 21102988 10.1093/sleep/33.11.1465 PMC2954696

[56] Lenz R.A., Baker J.D., Locke C., Rueter L.E., Mohler E.G., Wesnes K., Abi-Saab W., Saltarelli M.D. The scopolamine model as a pharmacodynamic marker in early drug development. Psychopharmacology (Berl). 2012; 220(1): 97–107. 21901320 10.1007/s00213-011-2456-4

[57] Dawson G.R., Iverson S.D. The effects of novel ChE inhibitors and selective muscarinic receptor agonists in test of reference and working memory. Behav. Brain Res. 1993; 57: 143–153. 8117420 10.1016/0166-4328(93)90130-i

[58] Chen Z., Xu A.J., Li R., Wei E.Q. Reversal of scopolamine induced spatial memory deficits in rats by TAK-147. Acta Pharmacol. Sin. 2002; 23(4): 355–360. 11931694

[59] Easton A., Sankaranarayanan S., Tanghe A., Terwel D., Lin A.X., Hoque N., Bourin C., Gu H., Ahlijanian M., Bristow L. Effects of sub-chronic donepezil on brain Abeta and cognition in a mouse model of Alzheimer’s disease. Psychopharmacology (Berl). 2013; 230(2): 279–289. 23783773 10.1007/s00213-013-3152-3

[60] Chuah L.Y., Chee M.W. Cholinergic augmentation modulates visual task performance in sleep-deprived young adults. J. Neurosci. 2008; 28(44): 11369–77. doi: 10.1523/JNEUROSCI.4045-08.2008 18971479 10.1523/JNEUROSCI.4045-08.2008 PMC6671517

[61] Chuah L.Y., Chong D.L., Chen A.K., Rekshan W.R 3rd, Tan J.C., Zheng H., Chee M.W. Donepezil improves episodic memory in young individuals vulnerable to the effects of sleep deprivation. Sleep. 2009; 32(8): 999–1010. 19725251 10.1093/sleep/32.8.999 PMC2717207

[62] Braida D, Paladini E., Griffini P., Lamperti M., Maggi A., Sala M. An inverted U-shaped curve for hepatylphysostigmine on radial maze performance in rats: comparison with other cholinesterase inhibitors. Eur. J. Pharmacol. 1996; 302(1–3): 13–20. 8790986 10.1016/0014-2999(96)00072-6

[63] Flood J.F., Landry D.W., Jarvik M.E. Cholinergic receptor interactions and their effects on long-term memory processing. Brain Res. 1981; 215(1–2): 177–185. 7260586 10.1016/0006-8993(81)90500-x

[64] Michaels R.L., Rothman S.M. Glutamate neurotoxicity in vitro: antagonist pharmacology and intracellular calcium concentrations. J. Neurosci. 1990; 10: 283–292. 1967639 10.1523/JNEUROSCI.10-01-00283.1990 PMC6570346

[65] Francis P.T., Webster M.T., Chessell I.P., Holmes C., Stratmann G.C., Procter A.W., Cross A.J., Green A.R., Bowen D.M. Neurotransmitters and second messengers in aging and Alzheimer’s disease. Ann. NY. Acad. Sci. 1993; 695: 19–26. 7902054 10.1111/j.1749-6632.1993.tb23021.x

[66] Greenamyre J.T., Young A.B. Excitatory amino acids and Alzheimer’s disease. Neurobiol. Aging. 1989; 10: 593–602. 2554168 10.1016/0197-4580(89)90143-7

[67] Van Dam D., De Deyn P.P. Cognitive evaluation of disease modifying efficacy of galantamine and memantine in APP23 model. Eur. Neuropsychopharmacol. 2006; 16(1): 59–69. doi: 10.1016/j.euroneuro.2005.06.005 16095884 10.1016/j.euroneuro.2005.06.005

[68] Tarragon E., Lopez D., Estrada C., Gonzalez-Cuello A., Ros C.M., Lamberty Y., Pifferi F., Cella M., Canovi M., Guiso G., Gobbi M., Fernandez-Villalba E., Blin O., Bordet R., Richardson J.C., Herrero M.T. Memantine prevents reference and working memory impairment caused by sleep deprivation in both young and aged Octodon degus. Neuropharmacology. 2014; 85: 206–214. doi: 10.1016/j.neuropharm.2014.05.023 24878242 10.1016/j.neuropharm.2014.05.023

[69] Schugens M.M., Egerter R., Daum I., Schepelmann K., Klockgether T., Loschhmann P.A. The NMDA antagonist memantine impairs classical eyeblink conditioning in humans. Neurosci. Lett. 1997; 224: 57–60. 9132691 10.1016/s0304-3940(97)13452-8

[70] Rammsayer T.H. Effects of pharmacologically induced changes in NMDA-receptor activity on long-term memory in humans. Neurobiol. Learn. Mem. 2001; 8: 20–25. 10.1101/lm.33701 PMC311354 11160760

[71] Korostenskaja M., Nikulin V.V., Kicic D., Nikulina A.V., Kahkonen S. Effects of NMDA receptor antagonist memantine on mismatch negativity. Brain Res. Bull. 2007; 72: 275–283. doi: 10.1016/j.brainresbull.2007.01.007 17452287 10.1016/j.brainresbull.2007.01.007

[72] Tariot P.N., Farlow M.R., Grossberg G.T., Graham S.M., Mc Donald S., Gergel I. Memantine treatment in patients with moderate to severe Alzheimer disease already receiving donepezil: a randomized controlled trial. JAMA. 2004; 291: 317–324. doi: 10.1001/jama.291.3.317 14734594 10.1001/jama.291.3.317

[73] Reisberg B., Doody R., Stoffler A., Schmitt F., Ferris S., Mobius H.J. A 24-week open-label extension study of memantine in moderate to severe Alzheimer disease. Arch. Neurol. 2006; 63: 49–54. doi: 10.1001/archneur.63.1.49 16401736 10.1001/archneur.63.1.49

[74] Bons N., Rieger F., Prudhomme D., Fisher A., Krause K.H. Microcebus murinus: a useful primate model for human cerebral aging and Alzheimer’s disease? Genes Brain Behav. 2006; 5(2): 120–130. doi: 10.1111/j.1601-183X.2005.00149.x 16507003 10.1111/j.1601-183X.2005.00149.x

[75] Languille S., Liévin-Bazin A., Picq JL., Louis C., Dix S., De Barry J., Blin O., Richardson J., Bordet R., Schenker E., Djelti F., Aujard F. Deficits of psychomotor and mnesic functions across aging in mouse lemur primates. Front Behav Neurosci. 2015; 9: 8:446 doi: 10.3389/fnbeh.2014.00446 25620921 10.3389/fnbeh.2014.00446 PMC4288241

[76] Schneider JS, Pioli EY, Jianzhong Y, Li Q, Bezard E. Effects of memantine and galantamine on cognitive performance in aged rhesus macaques. Neurobiol Aging. 2013; 34:1126–32. doi: 10.1016/j.neurobiolaging.2012.10.020 23158762 10.1016/j.neurobiolaging.2012.10.020

