# Supplementary material for: Effects of acute administration of donepezil or memantine on sleep-deprivation-induced spatial memory deficit in young and aged non-human primate grey mouse lemurs (Microcebus murinus)
Source: PLoS One. 2017 Sep 18;12(9):e0184822. doi: 10.1371/journal.pone.0184822 (PMC5602634; doi:10.1371/journal.pone.0184822)
Supplement: S1 Table — Raw data of young and aged animals before (day 1, D1) and after (day 2, D2) 8h sleep deprivation, with saline or two doses (0.1mg/kg [0.1] or 1 mg/kg [1]) of donepezil (DPZ) or memantine (MEM). (DOCX) [file pone.0184822.s001.docx]

| **Young Number of Errors** | | | | | | | | | |
| --- | --- | --- | --- | --- | --- | --- | --- | --- | --- |
| Saline D1 | Saline D2 | DPZ 0.1 D1 | DPZ 0.1 D2 | DPZ 1 D1 | DPZ 1 D2 | MEM 0.1 D1 | MEM 0.1 D2 | MEM 1 D1 | MEM 1 D2 |
| 4 | 4.5 | 2.5 | 1.5 | 5 | 2.5 | 3 | 8.5 | 9 | 2 |
| 2 | 3 | 6 | 2.5 | 4.5 | 2 | 3 | 6.5 | 1 | 4 |
| 2 | 5.5 | 7 | 0 | 1 | 2.5 | 5 | 0.5 | 6.5 | 5.5 |
| 1.5 | 2.5 | 2 | 1.5 | 1.5 | 3.5 | 6 | 7 | 4.5 | 0 |
| 3 | 3 | 1.5 | 6.5 | 7.5 | 3 | 2.5 | 4.5 | 3.5 | 7 |
| 3.5 | 4.5 | 6 | 1 | 4.5 | 3 | 3 | 5.5 | 4 | 1 |
| 3.5 | 5.5 |  |  | 2.5 | 8 |  |  | 4 | 5 |
| 1.5 | 2 |  |  |  |  |  |  |  |  |
| **Aged Number of Errors** | | | | | | | | | |
| 0.5 | 3.5 | 6.5 | 24 | 11.5 | 2.5 | 1 | 6 | 3.5 | 3 |
| 3.5 | 8 | 5.5 | 7 | 2 | 1.5 | 0.5 | 5.5 | 1 | 3.5 |
| 5.5 | 10 | 14 | 4.5 | 2.5 | 0.5 | 0.5 | 9.5 | 1 | 4 |
| 4 | 5 | 10 | 1.5 | 2.5 | 4 | 2 | 15.5 | 3.5 | 2 |
| 9 | 13 | 2.5 | 5 | 4.5 | 4 | 2.5 | 1.5 | 6 | 13.5 |
| 2.5 | 11 | 6 | 10.5 | 6 | 3.5 | 5 | 6 | 4.5 | 5 |
|  |  |  |  | 5.5 | 5.5 | 2 | 9 | 6 | 10 |
|  |  |  |  |  |  |  |  | 9 | 5 |
| **Young Latency** | | | | | | | | | |
| 393 | 213 | 300 | 201 | 78.5 | 312.5 | 249 | 152 | 316.5 | 136.5 |
| 556.5 | 563.5 | 426 | 600 | 364.5 | 600 | 373.5 | 483 | 206.5 | 404.5 |
| 585.5 | 551.5 | 373.5 | 124.5 | 195 | 317 | 379 | 67 | 391.5 | 200.5 |
| 180 | 282 | 146.5 | 123 | 435.5 | 145.5 | 553.5 | 425 | 485.5 | 134.5 |
| 234 | 472 | 390 | 346.5 | 429 | 498 | 429.5 | 393.5 | 370 | 495 |
| 330 | 425.5 | 507 | 328 | 276 | 377 | 194 | 431 | 269 | 139 |
| 573.5 | 600 |  |  | 5.5 | 127.5 |  |  | 95 | 600 |
| 432.5 | 327 |  |  |  |  |  |  |  |  |
| **Aged Latency** | | | | | | | | | |
| 10.5 | 264.5 | 371.5 | 541.5 | 257 | 37.5 | 261.5 | 411.5 | 555.5 | 198.5 |
| 205 | 99.5 | 346.5 | 353 | 382 | 600 | 79.5 | 42 | 130 | 362 |
| 104 | 87 | 577 | 459.5 | 437 | 66 | 35 | 234.5 | 67.5 | 106 |
| 224 | 179 | 518.5 | 223.5 | 505.5 | 88.5 | 391 | 511 | 525 | 503.5 |
| 375.5 | 343.5 | 493 | 182 | 88.5 | 143 | 445 | 600 | 167 | 252 |
| 244 | 273 | 111 | 111 | 264 | 102 | 574 | 65.5 | 426.5 | 360 |
|  |  |  |  | 561.5 | 224 | 114.5 | 109 | 431 | 251.5 |
|  |  |  |  |  |  |  |  | 476 | 556.5 |
| **Young Rank zone** | | | | | | | | | |
| 2 | 5 | 2.5 | 2 | 5 | 3.5 | 4 | 7 | 3.5 | 1.5 |
| 3 | 4 | 3.5 | 3.5 | 4.5 | 2.5 | 2.5 | 3.5 | 2 | 1.5 |
| 3 | 4 | 3.5 | 1 | 1.5 | 3 | 6 | 1.5 | 4.5 | 2.5 |
| 2.5 | 3.5 | 1.5 | 2.5 | 2 | 2 | 2 | 6 | 4.5 | 1 |
| 2.5 | 2.5 | 1 | 3.5 | 3.5 | 4.5 | 2.5 | 3.5 | 2.5 | 4 |
| 4.5 | 1.5 | 3.5 | 1.5 | 3 | 4 | 4 | 4 | 4.5 | 2 |
| 4 | 6.5 |  |  | 2.5 | 1.5 |  |  | 5 | 5 |
| 2.5 | 3 |  |  |  |  |  |  |  |  |
| **Aged Rank zone** | | | | | | | | | |
| 1 | 2 | 4 | 3 | 4.5 | 3.5 | 1.5 | 4 | 4.5 | 3 |
| 4 | 5.5 | 6.5 | 2 | 1 | 2.5 | 1.5 | 2.5 | 1 | 4.5 |
| 3.5 | 3.5 | 3.5 | 3 | 2.5 | 1.5 | 0.5 | 5.5 | 2 | 2.5 |
| 3 | 5 | 2.5 | 2 | 3.5 | 1.5 | 3 | 5 | 4.5 | 2.5 |
| 3.5 | 5 | 3 | 2 | 2.5 | 2 | 2.5 | 4 | 2.5 | 5.5 |
| 2 | 6.5 | 3.5 | 3.5 | 3 | 3 | 3.5 | 2.5 | 5.5 | 5.5 |
|  |  |  |  | 1.5 | 2 | 3.5 | 2 | 1.5 | 6.5 |
|  |  |  |  |  |  |  |  | 4.5 | 1 |
| **Young Repetitions** | | | | | | | | | |
| 0 | 0 | 0 | 0 | 0.5 | 0 | 0.5 | 2 | 3 | 0 |
| 0.5 | 0 | 0.5 | 0 | 1 | 0 | 0.5 | 0.5 | 0 | 1 |
| 0 | 1.5 | 2.5 | 0 | 0 | 0 | 1 | 0 | 1 | 1 |
| 0 | 0 | 0 | 0 | 0 | 0.5 | 0.5 | 2 | 1 | 0 |
| 0.5 | 0 | 0 | 1 | 1.5 | 0.5 | 0 | 0 | 0.5 | 1 |
| 0.5 | 1 | 0.5 | 0 | 0 | 0 | 0 | 0.5 | 0 | 0 |
| 0.5 | 0 |  |  | 0 | 3 |  |  | 0 | 0.5 |
| 0 | 0.5 |  |  |  |  |  |  |  |  |
| **Aged Repetitions** | | | | | | | | | |
| 0 | 0.5 | 1.5 | 15 | 6.5 | 0 | 0 | 1 | 0 | 0.5 |
| 0 | 4 | 1.5 | 1 | 0 | 0 | 0 | 1.5 | 0 | 1 |
| 0.5 | 3 | 6.5 | 1 | 0.5 | 0 | 0 | 3 | 0 | 0 |
| 1 | 5 | 3.5 | 0 | 0.5 | 0 | 0 | 7 | 0 | 0 |
| 3.5 | 5.5 | 0 | 0 | 0 | 0 | 0 | 0 | 1.5 | 5.5 |
| 0 | 4 | 1 | 2 | 1.5 | 0.5 | 0.5 | 0 | 0 | 0 |
|  |  |  |  | 0.5 | 0 | 0 | 2 | 1.5 | 3.5 |
|  |  |  |  |  |  |  |  | 1.5 | 1 |

Supplementary Table 1. Raw data of young and aged animals before (day 1, D1) and after (day 2, D2) 8h sleep deprivation, with saline or two doses (0.1mg/kg [0.1] or 1 mg/kg [1]) of donepezil (DPZ) or memantine (MEM).

Performance was assessed by the number of errors (entering the four limbs in an incorrect compartment), the latency (the total time required by the animal to reach the target), the rank of the target zone (two adjacent quadrants surrounding either side of the goal-box containing quadrant; the rank was measured by the number of errors to reach the target zone), and the number of repetition (entry in the same quadrant more than one time) during the testing. Number of errors, Rank Zone and Number of Repetitions have no units, these parameters express a number of events, while Latency is expressed in seconds.
